# Our Dental Law

**Published:** 1903-09-15

**Authors:** Henry K. Lathrop


THE
DENTAL REGISTER.

Vol. LVII. SEPTEMBER 15, 1903. No. 9.

### OUR DENTAL LAW.

BY HENRY K. LATHROP, JR., D.D.S.

Read before the Michigan State Dental Association, July 9, 1903.

My knowledge of dentistry is not by any means as great as I desire, but what I don't know about law would fill volumes. Just why your committee on program selected me to write a paper on this subject I am not quite sure, unless it was because I was for thirteen or fourteen years one of the unfortunates (as a member of the State Board of Examiners in Dentistry) that acted as a part of the machinery of the law. As I have intimated to you, I am no lawyer and, as a consequence, it will take very little time for me to tell you what I know about the law. I am going to be very brief, but perhaps even with my brevity and limited knowledge I may be able to arouse a discussion which will be far more valuable to the members of this association than this paper.

Why do we have dental laws? During the time I was a member of the Board I heard a great variety of opinions on this subject, most of them coming from one of three general classes; one, the people; another, the dental profession that support and comply with the requirements of the law; and the third, violators of the law made up of all sorts even those who do not comply with the law apparently from no other reason than "pure cussedness" and then down through all grades of violators, to the traveling mountebank. I speak of one class as violators from "pure cussedness," for they fail to register, although all that is required of them is to make an affidavit and pay the registration fee. These persons do an immense amount of harm on account of their example.

The consensus of opinion among the people seems to be that the law is intended entirely for their protection. This is quite a natural conclusion to come to, but who ever heard the people clamoring for legislation to protect them from the incompetent dentist, lawyer or doctor. The dentists who comply with the law think that it was passed to protect them while incidentally doing the same thing for the people. The majority of those practicing dentistry illegally seem to think that the law was passed to make a graft for a certain privileged class of dentists and to wipe them out of existence; that the law is all wrong; that it should be torn up, root and branch; that it is unconstitutional; that no one has a right to deprive another from earning a living, etc. Of course, we all know there is a large class of people who object to law of any kind. If they desire a drink of liquor they should be allowed to drink when and where they please; if they see anything they want they should be allowed to take it whether it belongs to some one else or not; if any one harms them they should be allowed to take vengeance in their own hands even to the extent of taking the life of the offender.

It matters little what law is enacted, there will soon be innumerable persons insisting that it interferes with their liberty. I think that feeling exists to a great extent in the minds of nearly every one. We all feel more or less antagonistic to the law whether it be human or divine. This reminds me of the Irish immigrant who had just arrived at Castle Garden from his native bog. On being asked by the inspector what his politics were, he said: "I dunno, but I'm agin the government."

My idea is that dentistry, as practiced to-day by the best element of the profession, has reached such a high plane that, to reach that plane, much is required of a man. The requirements of our best institutions for entrance to their dental departments are as great as for any of the other learned professions, or for entrance to the academic course. After entrance it requires years of study, practice and training before the student can get a diploma. This means that a man has long passed his majority and has expended a large amount of money, and must still expend more to equip himself for practice. After he has done all this he is fitted to render a service to the people that is not to be equaled by another person who has not done practically the same thing. So that it is no more than fair and just that the educated professional man and the people join together and ask the Legislature to protect him and them from any lower grade of dentistry.

Of course we all know that even after a man has prepared himself in this manner to practice dentistry, he will, if he takes the right course, become much more proficient and skillful from practice and experience. But if he is tempted by the glitter of the dollar to join the mighty army of the dental parlor men he will be sure, from the nature of that class of practice, to retrograde and become, very likely, an inferior practitioner to one who has had less preparation, but is conscientious and has a high aim. No amount of legislation will ever protect us or the people from the dental parlor man, for nearly all of the heads of those institutions become registered dentists before they open up. But the men in their employ are the ones that are hard to reach. "We employ none but experts in every department," is the way they are advertised. Just how to get at these "experts" is not so clear. I think our law is very strong in this regard and will call your attention to this point when I come to that section.

You all know that our Michigan law is quite simple as compared to the laws of many other States, but from what I can learn it is quite as effective as any of them. Our law has been amended twice since the original act was passed. The first amendment was a very great improvement, the second was well intended, I suppose, but it amounts to nothing. I have talked with quite a number of dentists in this State who seem to think that our law is very inefficient and could be greatly improved; I don't quite agree with them, although I think that some improvements could be made if all the conditions were favorable. I will run over the law hurriedly with you, if you have the patience, and point out what improvements have been made and what changes are desirable in my estimation.

In the old original law, Section 1 was very vague and difficult to work under; it said that a man must have a diploma from a reputable dental college. That word "reputable" was a very difficult one to define. Section 1 of the amendment law makes it very plain. It reads that a man must have a diploma from a reputable dental college with a course of instruction and practice equal or equivalent to the college of Dental Surgery of the University of Michigan. The old law did not require that a man should receive a certificate; the present law says that he must have a certificate of qualification from the Board, obtained either by presenting a diploma that is accepted by the Board or by passing a satisfactory examination.

I think Section 2 would be much more satisfactory if one change were made in it. It reads as follows:

> Said Board of Examiners shall be appointed by the Governor of this State, and shall consist of three practical dentists, who shall be regular graduates of a reputable dental college, duly incorporated under the laws of this State or some other State of the United States, or otherwise possess the necessary qualifications contemplated by this act.

This is all right in every respect if the appointments are made regardless of politics, but I am afraid many times they are made for political purposes. In many of the States the Governor is required to appoint members of the Board from a list of names that have been nominated by the State

Dental Association. If this association were empowered to furnish the Governor, say, ten names every time there was to be a new member appointed to the Board, and he were obligated to appoint one of those names, I think there there is little doubt that we should have a thoroughly-competent and honest Board. Of course, you all understand that this is absolutely essential, for, without such a Board, there might as well be no law regulating the practice of dentistry.

I can suggest no change in any of the next seven sections, but call your attention to changes that have been made and a few that might be made to the remainder of the law.

The old law was very defective in regard to all of the fees. For instance, the registration fee was only twenty-five cents, which was absolutely inadequate, as it did not furnish funds enough to pay the postage account of the Board. The present registration fee of three dollars does very well, but I think, in this respect, the Wisconsin law is better. It requires registration every year, the fee being one dollar which gives the Board an income sufficient to look after violators and, of course, makes much more work for the Board. The old law was also defective in regard to the examination fee. While the fee was the same, $10, it required the board to return it to the applicant if he or she were unsuccessful. This, as you can see, was a temptation to let the applicant through, as otherwise it was next to impossible for the Board to secure funds to pay its expenses. In the early part of my career on the Board I was often unable to get my per diem of three dollars and was for some years obliged to pay my own railroad and hotel expenses. Now it is quite different; the applicant,s fee is retained whether he passes the examination or not, and this gives the Board ample funds (with registration fees added) to pay all its expenses. There is one change that could be made in the law in regard to the funds that I think would be desirable. As the Board gets no help from the State treasury, I think it should not be required to turn over to the State Treasurer any funds in its treasury in excess of one hundred dollars which the present law requires it to do.

Section 9 requires the Board to keep an alphabetically arranged list of all licensed dentists. I think the Board should be required to publish this list at least once in two years.

Many laws are enacted that amount to very little, and are ineffective because there is no penalty of any consequence attached to them. This is not the case with our dental law, for the penalty is very severe for violators of it if they are convicted. It reads as follows:

> Section 12. Any person who shall practice dentistry in this State, in violation of the provisions of this act, shall be deemed guilty of a misdeameanor, and upon conviction thereof, shall be fined not less than twenty-five dollars, nor more than one hundred dollars, or sentenced to imprisonment in the county jail for a period not exceeding ninety days, or both, such fine and imprisonment (is) in the discretion of the court. Provided, That nothing in this act shall be construed so as to interfere with physicians and surgeons in their practice as such.

But *conviction*, there is where the rub comes. It is absolutely necessary to get a conviction before you get the penalty, and convictions are hard to get because what is everybody's business is nobody's business. It is almost impossible to find any one who will make a complaint and follow the case up and prosecute it vigorously to a finish. Once in a while a spasmodic effort will be made by a local dental society to prosecute all the violators of the law in the community, but it is a great deal of trouble, and in most cases the work will nearly all fall on one or at most two men. And they soon become tired of the task for many reasons. Besides taking considerable time it soon makes them a target for all sorts of abuse, not only from the violators but also from their friends and fellow-townsmen who ascribe the prosecution to jealousy or a thousand and one things to such an extent that in some cases I have known the complainants to be obliged to leave town. But I want to tell you, and in the most emphatic manner, that if complaints are not made and the law enforced there might as well be no law, for it will become a dead letter and a mere farce and the violators will simply laugh at you.

Section 13 is the one I referred to earlier in the paper. While it appears to be very strong it is where we have trouble with these so-called dental parlor experts. However I must confess that I can not suggest a change that I think would be an improvement. The section reads:

For the purpose of instruction students may be employed to assist in dental offices, and in the College of Dental Surgery of the University of Michigan, under the immediate observation and advice of the legal proprietors and professors thereof, but no person not legally qualified and registered under this act shall assume the charge and management of any dental office, or the responsibllity of deciding upon or the doing of dentistry at any private residence or elsewhere.

That seems very strong and it would seem almost impossible to evade its provisions, but if you arrest one of these same men the proprietors of the institution will go upon the stand at the trial and swear that the said expert did not have the responsibility or decide upon what should be done in this particular case for which he has been arrested. And even if you have the patient there as a witness and he swears that as far as he knows no one else had anything to do with the case, the proprietor will swear that the respondent consulted with him about the case and the respondent will swear to the same thing. Perhaps some of you can suggest a change that will accomplish the end we desire.

The last amendment to the law was the addition of another section which reads as follows:

Section 15. The Secretary of said Board shall, on the first day of June of each year hereafter, file an annual report with the Secretary of State, showing the number of applicants for examination, the number who passed said examination and received a license to practice, the amount of fees received from the applicants from such licenses granted and the amount received by each member for his expenses and services.

This is the one of which I spoke at the beginning and which I suppose was intended to be a great improvement. The idea was correct enough.

It was designed to make the Board report to the Secretary of State. One might think at first glance that the Board must give a full report and might be held to giving a full accounting, but such is not the case. In the first place this section requires the board to report the number of applicants for examination, the number who passed and the amount of fees received from those that passed. There is nothing said about money received from those who did not pass; nothing said about money received from temporary licenses; nothing said about reporting money received from registration. This last item alone amounts to something like $200 a year now or even more. If this section had required a full accounting in every particular, as to moneys received, from any and every source, and in fact a full report of all the business done, it would have done just what was expected of it.

You might conclude from what I have said and from the things I have called your attention to, that our law is very defective. But this is not so; most of the defects are of minor importance, especially if we have a thoroughly competent and honest Board. With the exceptions of the prosecutions a Board of this kind can do very effective work. The prosecutions are really no part of their business, and they should not be called on for anything in that line, except to give their testimony in court as it is always necessary for them to do in these cases. It would be a very admirable thing if we could have some one to look after that part of the business, as I understand the druggists do in similar cases. The most important thing, in my estimation, is the manner in which the Board is appointed.

There has been a great deal of talk lately about having certificates of one State good in every other State; that it is absurd that a man coming here from New York or from Maine or from any other State, with a certificate entitling him to practice in his own State, should be prevented from practicing here without an examination. I will admit that, at the first glance, it does seem absurd; but when one becomes familiar with the lax methods of the boards in some other

States, one can readily see what would be the result. Many seem to think that no one should be allowed to practice without passing an examination before the State Board, but that, I think, is a mistake. I think our law is perfect in that respect. When a man has taken the course at our own State University and has received his diploma, it should be a guarantee that he is competent to practice and all that should be required of him is the payment of a registration fee and the making of an affidavit that he is the man mentioned in the diploma. As to other colleges the law makes the Board the judge whether they are up to the standard required.

I have pointed out what seem to me to be about the only defects in our law. They are not many and while I should like to see those few defects remedied, I should want to be very sure that new defects are not to be added or some of the best features of the present law destroyed. I mean that while it would be quite possible for some man who has had a long experience in the administration of dental laws, assisted by an able lawyer, to draw a bill that would be about all that any human law could be; but after it had gone through both houses of the Legislature I don't believe the men who drew it would recognize it. So I say, beware of tinkering with a fairly good law.

